# The Use of Microneedling With Exosomes in Dermatology: A Systematic Review

**DOI:** 10.1111/jocd.70881

**Published:** 2026-04-24

**Authors:** Navnit Kaur Dhaliwal, Thurkga Moothathamby, Balamrit Singh Sokhal, Maria‐ Angeliki Gkini

**Affiliations:** ^1^ School of Medicine and Dentistry Queen Mary University of London London UK; ^2^ Wolfson Institute of Population Health Queen Mary University of London London UK; ^3^ Royal Stoke University Hospital University Hospitals North Midlands Stafford UK; ^4^ School of Medicine Keele University Keele Staffordshire UK; ^5^ Department of Dermatology The Royal London Hospital London UK

## Abstract

**Background:**

Microneedling combined with exosome therapy has growing clinical relevance in dermatology. This systematic review will critically evaluate current evidence on the combined use of microneedling and exosomes in a number of dermatological diseases.

**Method:**

A systematic literature search was conducted using terms related to exosomes and microneedling using 3 databases in accordance with the Preferred Reporting Items for Systematic Reviews and synthesis (PRISMA) without meta‐analyses (SWiM) guidelines. Studies were appraised using the JBI (Joanna Briggs Institute) Critical Appraisal tool.

**Results:**

Two hundred and fifty‐six unique references were identified, and after screening, 10 studies were eligible for quality appraisal, after which 8 studies were selected for inclusion in the final narrative synthesis. Sample size of the studies ranged from 3 from 60 participants. The efficacy and side effect profiles of exosomes and microneedling were evaluated in the following dermatological conditions: androgenetic alopecia, skin aging, hyperpigmentation, active scarring, and enlarged pores.

**Conclusion:**

Microneedling and exosomes hold great promise in a range of dermatological conditions. This study highlights that more evidence is required before we can ascertain the safety profile and efficacy profile of microneedling and exosomes. Further investigation with longer follow‐up periods and larger sample sizes is necessary to determine the long‐term safety profile and effectiveness of this treatment.

## 1 Introduction and Background

The recent decades have witnessed a marked rise in the use of microneedling procedures across both aesthetic and general medical dermatology. Whilst microneedling can be utilized as a stand‐alone treatment, it has recently evolved with combined use of exosome therapy. Microneedling, also known as percutaneous collagen induction therapy [1], is a medical procedure that involves inserting hypodermic needles directly into the region of the dermatological defect e.g., acne scar. The current general consensus on the mechanism of action proposes that the insertion of these needles causes microchannels to be formed and this disrupts the collagen and architectural structure of the tissue underneath the skin, inducing a so‐called “controlled injury” or “therapeutically induced trauma” [1]. This then induced the body to initiate a repair pathway which stimulates the production of new collagen and elastin, as well as the release of growth factors and neovascularisation in the region; this process occurs over several weeks to months [1]. This ultimately leads to the creation of new tissue which restores volume in the skin depression [1], as well as healing the underlying skin pathology.

Exosomes are small, extracellular vesicles that play an essential role in cell to cell signaling and molecular cargo transport [2]. These are released by a number of different cell types in the body [2]. Exosomes are primarily composed of internal contents (such as nucleic acids, proteins, and metabolites [3]) surrounded by a lipid bilayer membrane. The lipid bilayer is enriched with membrane proteins and facilitates high‐specific target fusion with recipient cells, enabling efficient cargo delivery [2]. Engineered exosomes offer vast clinical versatility, as their cargo can be selectively modified to include specific therapeutic agents [2, 3]. Exosomes are actively used in conjunction with microneedling, resulting in therapeutic outcomes through parallel but distinct mechanisms of action.

Microneedling combined with exosome therapy has demonstrated growing clinical relevance in dermatology. This systematic review will critically evaluate current evidence on the combined use of microneedling and exosomes in a number of dermatological diseases.

## 2 Methods

### 2.1 Search Strategy and Study Selection

**FIGURE 1:**
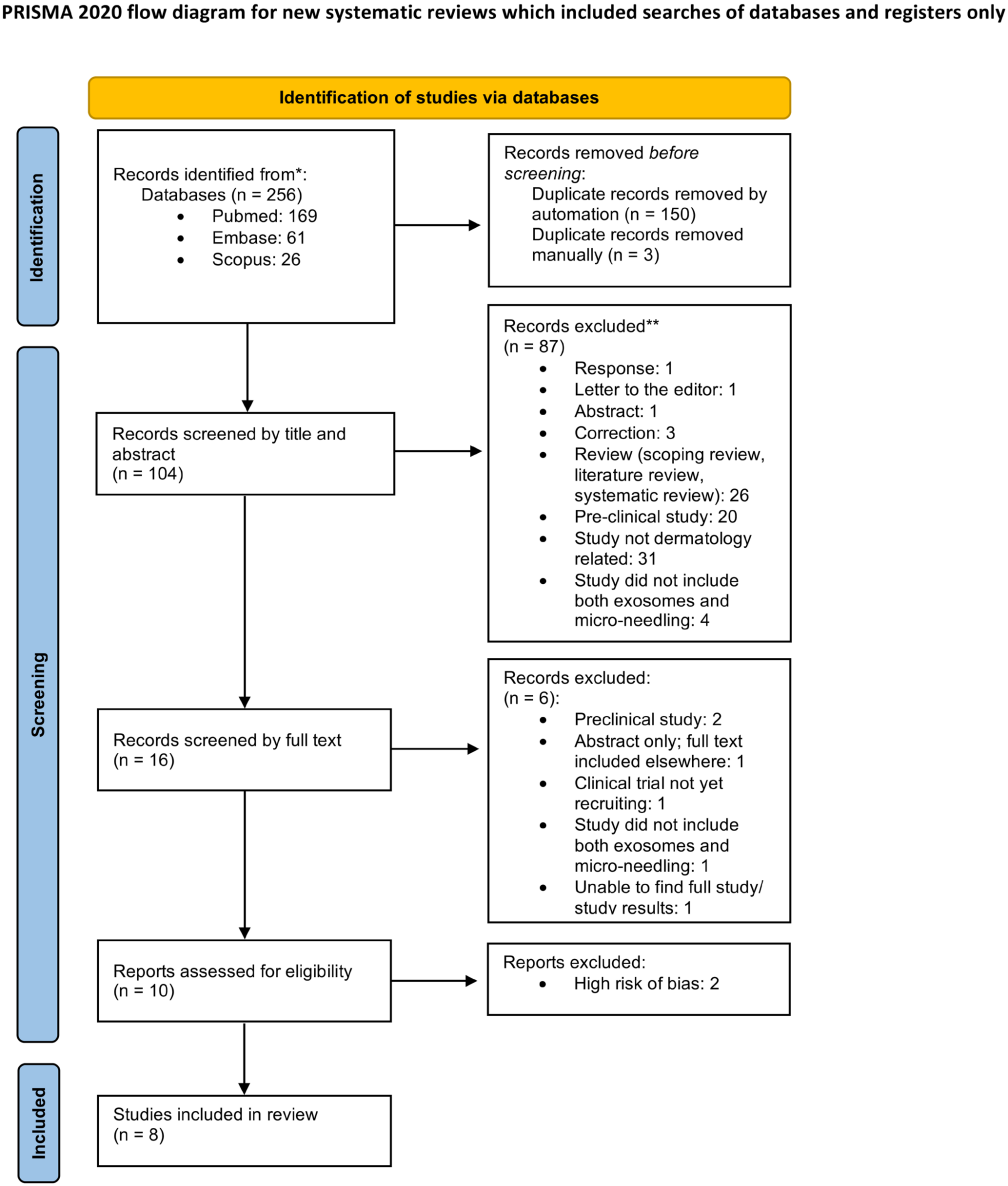
PRISMA flow diagram of the systematic review.

The protocol for this review was registered with the International Prospective Register of Systematic Reviews (PROSPERO, CRD420251102391). This systematic review follows Preferred Reporting Items for Systematic Reviews and Meta‐Analyses (PRISMA) guidance (Figure 1) [4]. Pubmed, Embase (Ovid) and Scopus databases were queried from inception to June 2025 by one reviewer (NK). The search strategy utilized both database subject headings and text work searching in the title and abstract using terms for Exosomes AND microneedling, exosomes WITH microneedling and exosomes WITH microneedling AND dermatology.

### 2.2 Inclusion and Exclusion Criteria

All studies that involved the use of both microneedling with exosomes in dermatological conditions in English were included. The study design types that were included were retrospective studies, case series, case studies, clinical trials, and prospective studies. Review articles (scoping review, literature review, and systematic review), animal studies, and preclinical studies were excluded.

### 2.3 Screening and Data Extraction

All references were imported into a systematic review management software and de‐duplicated by automation and manually by one reviewer, ND. Two reviewers, ND and TM, independently then screened all titles and abstracts against inclusion criteria, excluding references that did not meet these criteria. ND and TM independently screened the full texts of the articles against the inclusion and exclusion criteria. In cases where only the abstracts were identified, efforts were made to locate the full article through supplementary searching. If the full text was not found, the study was excluded. If the full text was found, then it was screened following the exclusion and inclusion criteria. When multiple abstracts or conference abstracts pertained to the same study, only one version was included in the analysis, and the remaining abstracts were excluded as duplicates.

Two reviewers, NK and TM, independently extracted data from full‐text articles. The following data were extracted: study type/design, dermatological conditions being treated, number of participants, age range, brief description of intervention, outcome, and adverse effects. Disagreements throughout the screening and data extraction process were resolved by discussion. A third independent reviewer (BSS) provided a resolution if disagreements remained.

### 2.4 Quality Evaluation and Risk of Bias

The quality of each study was assessed using the Joanna Briggs Institute (JBI) Critical appraisal tool. Given the vast differences in methodology of the included studies, the JBI critical appraisal tool was utilized to facilitate a consistent and reliable evaluation of study quality and bias. To ensure accurate evaluation, the appropriate JBI appraisal tool was selected based on the design of each study, which included randomized controlled trials (RCTs), case series and quasi‐experimental [5, 6, 7].

Two team members carried out an independent screen of the selected articles. Discrepancies in scoring were resolved through discussion. If the total percentage score was 70% or above, the study would be included. Studies below 70% would not be included due to high risk of bias. This cut off was established via general consensus by the researchers.

### 2.5 Data Synthesis and Analysis

Due to anticipated substantial heterogeneity in outcome measures, study designs and intervention protocols a meta‐analysis was not conducted. Instead a qualitative synthesis was utilized to interpret and compare results using the synthesis without meta‐analysis (SWiM) guidance and Cochrane Handbook [8, 9]. Results were grouped and discussed according to the dermatological condition being treated in each study.

### 2.6 Ethical Considerations

Ethical approval was not required as this systematic review analyzed and interpreted data from existing studies. No new studies involving human or animal tissue or participants were conducted.

## 3 Results

### 3.1 Screening and Quality Evaluation Outcome

Two hundred and fifty‐six unique references were identified, and after screening, 10 studies were eligible for quality appraisal (Table 1). One point was given for yes, 0 points was given for no or not clear to each question/domain. N/A was written if this was not applicable in the study and therefore the total score was reduced for that study. If the total percentage score was 70% or above, the study would be included.

**TABLE 1:** Critical appraisal of studies.

Study name	Q1	Q2	Q3	Q4	Q5	Q6	Q7	Q8	Q9	Q10	Q11	Q12	Q13
Lee et al., 2024 [10]	1	0	0	1	1	1	1	1	1				
Park et al., 2023 [11]	1	0	1	N/A	0	0	1	1	1	1	1	1	1
Estubinan et al., 2025 [12]	1	1	1	1	1	1	1	1	0				
Proietti et al., 2024 [13]	1	1	1	0	1	1	1	1	1				
Theodorakopoulou et al., 2024 [14]	1	N/A	N/A	0	1	N/A	1	1	1	1			
Wang and Gao et al., 2023 [15]	1	1	1	1	0	0	1	1	1				
Wan and Pamela et al., 2025 [16]	1	1	1	0	0	1	1	1	0	1			
Wan et al., 2025 [17]	1	1	1	0	0	1	1	1	1	0			
**Nadeem et al., 2024** [18]	**0**	**0**	**0**	**1**	**0**	**0**	**1**	**0**	**1**	**1**	**1**	**1**	**1**
**Wang at el, 2022** [19]	**1**	**N/A**	**N/A**	**0**	**1**	**1**	**N/A**	**1**	**0**				

Studies by Lee et al., Estubian et al., Proietti et al., Wang & Gao et al., Wang et al. and Theodorakopoulou et al. were screened using the JBI qausi experimental tool. Studies by Park et al. and Nadeem et al. were screened using the JBI randomized control trial tool. Studies by Wan & Pamela et al. and Wan et al. were screened uring the JBI case series tool. Out of 10 studies, 2 studies (in red) were excluded, as they did not meet the appraisal threshold of 70%. Eight studies were analyzed for discussion.

### 3.2 Study Characteristics

From 8 studies, there was a total of 171 patients. Data were from South Korea (*n* = 3), the United States (*n* = 1), Italy (*n* = 1), Greece (*n* = 1), and China (*n* = 1). In one study, the location was not stated. Studies investigated the use of microneedling and exosomes in the following conditions: Androgenetic alopecia, skin aging, melasma, hyperpigmentation, enlarged pores, active acne, and atrophic scarring. Study characteristics were summarized in Table 2.

**TABLE 2:** Study characteristics.

Name	Study design	indication	No. of participants	Age range	Brief description of intervention	Outcome measurements	Adverse effects
Lee et al. 2024	24 week open‐label prospective study with a single‐arm design	Androgenetic alopecia	30	18–60	Participants were treated with freeze‐dried exosome solution	Hair density changes, expert photographic assessment and patient satisfaction score	Slightly tingling (*n* = 5), temporary erythema after application (*n* = not mentioned)
Park et al., 2023	12 week prospective, randomized split‐face comparative study	Skin aging	28	43–66	Treated 28 participants with human adipose stem cell‐derived exosome plus microneedling on one side of the face versus saline plus microneedling on the other side of the face	Standardized photography using the Global Aesthetic Improvement Scale (GAIS), skin wrinkles were assessed using (PRIMOS Premium), elasticity (Cutometer), hydration (Corneometer) and hyperpigmentation Mark‐Vu imaging system, and with additional histopathological evaluation	Transient erythema, oedema and petechiae were observed‐resolved within 1 week
Estubian et al., 2025	Investigator‐blinded, split‐face trial	Skin aging	15	44–68	Participants received three full‐face radiotherapy microneedling treatment at 4‐week intervals, with platelet‐rich fibrin matrix (PRFM) applied to the right side and exosome products to the left side, followed by specific post‐treatment care regimens for each side over 10 days.		
Proietti et al., 2024	Monocentric, observational pilot study	Melasma	20	18–69	Each subject received microneedling treatment followed by application of exosomes to the area of melasma. The interventions were performed at 4‐week intervals, up to a maximum of 5 sessions.	mMASI (modified Melasma Area and Severity Index), The Global Aesthetic Improvement Scale and tolerability was measured.	Mild erythema and burning sensation reported by all patients, but was otherwise reported as well tolerated.
Theodorakopoulou et al., 2024	Open‐label, uncontrolled study	Hyperpigmentation	12	Mean age 46.64 ± 13.05 years	Topical application of Rose Stem‐Cell derived Exosomes (RSCE) with microneedling and a 20 min LED light with RSCE‐infused mask treatments were performed in 3 week intervals.	Global Aesthetic Improvement Scale (GAIS), Dermatology Life and Quality Index (DLQ), and melasma Qaulity of Life Scale (MELASQoL) scores were measured.	No serious adverse effects recorded. Treatment related adverse effects were erythema for mild oedema and pruritus and pain during the procedure.
Wang and Gao et al., 2023	Clinical application study	Melasma	60	18–60	Patients were split into 4 groups: Group A (Nonablative fractional laser + saline), Group B (microneedling + exosomes), Group C (Nonablative fractional laser + exosomes), and Group D (plasma + exosomes), with treatments applied to the entire face. Results of group B only were discussed.	Melasma Area and Severity Index (MASI) score, improvement rates, physician global assessment and patient satisfaction.	Pain and sensitivity to stimulation (*n* = 1) and lingering of red spots from the microneedles (*n* = 2)
Wan and Pamela et al., 2025	Case series	Active acne, PIH and atrophic scarring	3	28–51	Each patient received one session of microneedling followed by topical Lactobacilus derived exosome (LDE) therapy and was followed 2 months after.	Acne severity, hyperpigmentation and atrophic scarring parameters were assessed. Patient satisfaction was also assessed.	Transient erythema (*n* = 1)
Wan et al., 2025	Case series	Enlarged pores	3	32–55	Each patient received microneedling followed by topical application of stem cell‐derived exosomes administered in 3 sessions at 4‐week intervals.	Standardized photographs were evaluated using GAIS scale at 12 and 22 weeks.	No adverse effects were noted.

### 3.3 Efficacy of Microneedling and Exosomes in Androgenetic Alopecia

One study [10] assessed the use of combined treatment of microneedling and exosomes for androgenetic alopecia. Change in hair density was measured at baseline, at 12 weeks and at 24 weeks. At baseline the hair density was 158.03/cm^2^ and it increased to 166.14/cm^2^ (*p* < 0.001) at 24 weeks. This was a mean increase of 8.11/cm^2^ in 24 weeks. A global photographic assessment was also conducted. This was conducted by 2 independent assessors that used a 7 point scale (−3 to +3) [10]. The images were taken in the light, angle and position with the same camera at baseline, week 12 and week 24. Evaluations at week 12 indicated that 6.90% of subjects experienced slight deterioration, 58.62% of patients had no change, 31.03% showed minor improvements and 3.45% showed marked improvements. At week 24, these proportions shifted to 10.34%, 37.93%, 41.38% and 10.34% respectively. All results were statistically significant (P‐value = 0.023 at week 12, 0.004 at week 24) [10].

Subjective assessment by the patient was also analyzed using the same 7‐point scale. At week 24, significant improvements from baseline were observed in general scalp appearance (30.72%, *p* < 0.001), subjective hair density satisfaction (52.72%, *p* < 0.001), daily hair loss (55.17%, *p* = 0.003), and hair thickness (58.62%, *p* < 0.001). Additionally, 13.79% of participants reported improved satisfaction with hair growth (*p* = 0.002).

### 3.4 Efficacy of Microneedling and Exosomes in Skin Aging

Two studies [11, 12] assessed the use of efficacy of microneedling and exosomes in skin aging. Both studies conducted a split‐face trial; different parameters were measured in both studies. In the study conducted by Park et al. [11], the measured Global Aesthetic Improvement Scale (GAIS) was improved by microneedling and exosomes. At the final follow‐up visit (week 12), 46% of patients had a GAIS score of 3, 14% scored 4, and 14% scored 5 for the exosome and microneedling side (*p* < 0.05). In the control group, 46% scored 3, 7% scored 4, and 7% scored 5 for the control side (grade 1 = worsened, grade 2 = unchanged, 3 = improved, 4 = much approved, and 5 = very much approved). Skin wrinkle improvement was based on 3 parameters, which were average roughness, the maximum height of the roughness profile, and maximum height of the roughness. Reduction was 12.4%, 14.4%, and 13.4%, respectively, on the exosome and microneedling side. In contrast, these were 6.6%, 6.8%, and 7.1%, respectively, on the control side. This shows that the combination of exosome and microneedling had a statistically significant improvement in wrinkle reduction (*p* = 0.031, 0.008, and 0.007, respectively). In terms of skin elasticity, at week 12, skin elasticity increased by an average of 11.3% from baseline on the exosome and microneedling side but decreased by 3.3% on the control side (*p* = 0.002). Skin hydration saw an improvement of 6.5% compared to 4.5% for the exosome and microneedling vs. control group (*p* = 0.037). Reduction in skin pigmentation was 9.9% on the exosome compared to 1% on the control side (*p* = 0.044). Histological analysis at 12 weeks revealed that while both treatments increased collagen density, elastic fibers, mucin deposition, and new collagen synthesis compared to baseline, these improvements were greater on the exosome and microneedling‐treated side than the control side [11].

In the study conducted by Estupinan et al. [12]. A split face trial was conducted where the right side was applied with radiofrequency microneedling and plasma‐rich plasma (PRP), whereas the left side was applied with microneedling and exosomes. At 5‐point scale was used to assess the overall skin appearance, wrinkling dyschromia, erythema and texture: 0 (lowest quality/worst experience) to 5 (highest quality/best appearance). The baseline score 2.12 and with treatment with exosomes and microneedling was 2.68 and 3.14 at 3 and 6 month follow‐up visits, respectively [12]. Photo‐aging scores improved by 37% at 3 months and 22% at 6 months compared to baseline on the Griffiths scale [12]. 10/15 participants had biopsies taken at baseline, 3 months and 6 months. Histological analysis showed an increase in collagen I, glycosaminoglycans and collagen remodeling compared to baseline [12].

### 3.5 Efficacy of Microneedling and Exosomes in Melasma and Hyperpigmentation

Hyperpigmentation is where a region of skin becomes darker than the surrounding areas of skin. Hyperpigmentation can be caused by a number of different conditions such as Melasma, Post Inflammatory Hyperpigmentation (PIH), acne scarring, and photo‐aging. Two studies looked at the efficacy of microneedling and exosomes in hyperpigmentation [13, 14]. The study by Proietti et al. [13] focused on melasma, whereas the study by Theodorakopoulou et al. [14] focused on hyperpigmentation caused by solar lentigines, melasma, PIH, and/or periorbital hyperpigmentation.

In the proietti et al. study, the Global aesthetic improvement scale (GAIS) score after the final session, 20% of patients reported “much improved outcome” and 70% of patients reported “improved” outcome. However, the GAIS score for in the Theodorakopoulou et al. study at the final session, 58% of patients rating their conditions as “very much improved” and 42% as “much approved” (*p* = < 0.007) when treated with exosome and microneedling.

The mMASI (modified Melasma Area and Severity Index) score for the Proietti et al. study showed that 88.9% of participants went from moderate (at baseline) to mild score (study end point). 10% of participants showed no change [13]. The Theodorakopoulou et al. study measured pigmentation reduction via 4 parameters: superficial pigmentation (sp), percentage of deep pigmentation (dp), percentage of skin redness (sr), and percentage of non‐wrinkled skin (nw) using a 3D facial analyzer. By week 12, superficial dark spots reduced from 39.75% ± 8.9% to 26.8% ± 5.9% (*p* = 0.000). By week 12, deep pigmented lesions reduced from 45.33% ± 10.82% to 29.42% ± 7.19% (*p* = 0.000). Skin redness reduced from 47.17% ± 10.41% to 39.83% ± 10.36% (*p* = 0.000). By week 12, skin quality and wrinkles improved from 52.3% ± 12.78% to 58.67% ± 12.05% (*p* = 0.000).

### 3.6 Efficacy of Microneedling and Exosomes in Active Acne, Atrophic Scarring and PIH

Three cases in a case series looked at the effect of utilizing exosomes and microneedling in exosomes when patients had combined active acne, strophic scarring and PIH [16]. All patients in the case series had a single microneedling session with a topical application of exosomes. The average improvement for all 3 cases in the investigator global scale (0 being clear skin and 4 being severe acne) improved from baseline to final results at month 2. From 3.34 to 1.34. The PIHASI (Post‐Inflammatory Hyperpigmentation Area and Severity Index) measures the dark spots left behind after acne scar. The average improvement between the 3 cases was 12 and 6.67 on the PIHASI scale. Scarring was measured by the Goodman and Baron score. This was reduced from 3.34 to 2.34 across all 3 cases. The patient satisfaction score after the treatment was an average of 8.6/10 [16].

### 3.7 Efficacy of Microneedling and Exosomes in Enlarged Pores

Three cases in a case series assessed the use of exosomes and microneedling in reducing pores [17]. Three sessions were conducted in 4 week intervals. The GAIS score was used to assess outcomes. GAIS scores ranged from 1 (worse) to 5 (very much approved). Patient satisfaction was reported using a 4 point scale (0: not satisfied; 1: slightly satisfied; 2: satisfied; 3: very satisfied). Measurements were taken at 12 weeks and 24 weeks post final treatment. Patient satisfaction averages across all 3 cases were 2.67 and 3 weeks 12 and 24 respectively. Average GAIS score was 4.00 and 4.32 respectively.

### 3.8 Adverse Effects of Microneedling and Exosomes

Across all studies [10, 11, 12, 13, 14, 15, 16, 17], no patient discontinued the study due to adverse effects. In 2 studies, the adverse effects resolved under 1 week [11, 12]. In one study, the adverse effects resolved within 24–72 h [17]. In 3 studies, the adverse effects were resolved but the time frame was not stated [10, 13, 14]. In one case in a case series, no adverse effects were reported [16]. Common adverse effects included: slight tingling, mild erythema, oedema, petechiae, mild burning sensation, mild pruritus and pain during the procedure [10, 11, 12, 13, 14, 15, 16, 17]. One case reported one patient that experienced sensitivity to stimulation and two cases of excessive lingering of red spots post‐procedure [15]. Adverse effects were not reported in 1 case in one study [16].

## 4 Discussion

The combined use of exosomes and microneedling has various different uses in the field of dermatology, the efficacy of which varies depending on the clinical use. In terms of androgenetic alopecia, one study showed there was a hair density by 8.11cm^2^ in 24 weeks [10]. Furthermore, GAIS scale improvement at 24 weeks 41.38% of patients had minor improvements, 10.34% showed marked improvements, and 37.93% of patients saw no change [10]. This suggests that whilst exosomes and microneedling are promising for promoting hair growth, it shows that at this stage in time, their results are modest. This study only mentioned patients' characteristics as a group; individual characteristics of the patient were not mentioned. This would have been useful to include to enable a more detailed understanding of patients within the data e.g., if a certain type of AGA responded better than others.

Efficacy of exosomes and microneedling for aging was assessed by two studies [11, 12]. Across both studies, there was consistency of improvement in different parameters of skin aging, which included: GAIS, wrinkle roughness and height, skin elasticity, pigmentation, photoaging and overall skin appearance. These improvements were statistically significant. This suggests that perhaps exosomes and microneedling are efficacious for aesthetic use; however, the population's study was small and the follow‐up time was limited; further research is warranted.

Efficacy of microsomes and microneedling was also explored in the context of hyperpigmentation. Patient reported outcomes were positive across both studies [13, 14]. 100% of patients from the Theodorakopoulou study and 90% of patients from the Proietti study showed improved or above e.g., very much improved or much approved results in terms of hyperpigmentation. The study endpoints were 12 weeks for the Theodorakopoulou study and between 4 and 5 months for the Proietti study. Both studies did not do 6 month or 1 year follow up, and it was not possible to know if the results lasted after the final treatment session [13, 14].

Efficacy of microneedling and exosomes were also studied in their use in active acne, strophic scarring and PIH [16]. A case series highlighted that microneedling and exosomes was effective in improving a number of different parameters such as: active acne, scarring and post‐inflammatory after a single microneedling and exosome session. Most importantly the PIHASI score was reduced by nearly half from 12 to 6.67 [16]. This was measured at the two‐month endpoint. Subjective satisfaction scores by the patients averaged 8.6/10, which highlights they were pleased with their outcome [16]. However the sample size was small and there was limited follow up time in seeing whether the results sustained through to a longer period of time [16].

Efficacy of microneedling and exosomes for reducing treatment for pore size was effective as demonstrated by a case series of 3 patients [17]. There was an improvement across all parameters such as GAIS score and patient satisfaction. This improvement was sustained from 12 weeks (study end point) and at 24 weeks. This suggests that not only is the combination of microneedling and exosomes useful in reducing pore size but the effect is sustained over a longer period of time [17]. It would be useful to have a follow up of these patients at 1,5 and 10 years to see if the effects are sustained.

Across all studies [10, 11, 12, 13, 14, 15, 16, 17, 18, 19], the adverse effects were mild and temporary. There were, however, cases of patients reporting excessive lingering post procedure and excessive pain. This may suggest that microneedling and exosomes are safe in the short term; however, more robust longer‐term data is required with larger populations to confirm these findings and ensure long term safety. Whilst in these studies, exosomes and microneedling were considered generally safe, there is evidence in the literature that suggests there are complications such as delayed onset granulomas [20], post‐inflammatory hyperpigmentation [21], and anaphylaxis [22]. It is important to note that these complications occurred when the exosomes were injected rather than micro‐needled. Furthermore, the complications could be a result of exosome preparation itself. Therefore, it is vital to be aware that life‐threatening complications can occur.

There were many strengths with this paper. Firstly, this systematic review adhered to the PRISMA guidelines, ensuring a rigorous methodology. Secondly, two independent reviewers screened the abstracts and full text to minimize bias. Thirdly, 3 major databases were utilized for the search strategies: Pubmed, Embase and Scopus, which ensured a comprehensive review and that as many relevant studies as possible were included.

There were limitations in this study. The sample sizes were small, and the available evidence was largely derived from small‐scale case studies and split‐face trials conducted in single‐centre clinics. Additionally, the follow up periods were short, with no participants monitored beyond 5 years. Furthermore, the results of this systematic review may have been influenced by publication bias, as studies with statistically insignificant or inconclusive findings are less likely to get published [23]. This could have contributed to less selective availability of research, leading to the overestimation of treatment efficacy.

The use of microneedling and exosomes has significant clinical implications in dermatology. Microneedling causes controlled micro‐injuries, which then also allow penetration of exosomes; both of which serve to promote skin regeneration and collagen synthesis. This combination promotes a shift towards regenerative medicine, whereby aging, scarring, and pigmentation can be treated and prevented with faster recovery and minimal side effects. Future directions should aim to explore the use of microneedling and exosomes in a more robust manner such as clinical trials. These trials should be conducted over a number of years, and this would generate more reliable and robust results over a long period of time such as over 5 or 10 years. This would serve to establish if the efficacy and safety profile of exosomes with microneedling is sustained.

The exosomes utilized in these studies were obtained from either human cell tissue, plant cell tissue, or from bacterial cell lines. The Lee et al. study isolated and purified their exosomes from human adipose tissue from a healthy donor [10]. This was approved by the institutional review board of CHA University medical centre, Republic of Korea, and was in line with Korean Ministry of food and drug safety guidelines [10]. All procedures, including cell isolation and all subsequent extraction processes, were conducted entirely in their laboratory. The study by Wang et al. extracted their exosomes from umbilical cord tissues that were voluntarily donated by healthy women from the obstetrics and gynecology department at Fujjan Medical University Union Hospital [15]. All patients were consented, and consent forms were signed [15]. The human umbilical cord mesenchymal stem cells were isolated, purified, and processed to then extract the exosomes [15]. Each batch was tested before being administered to patients [15].

The following studies utilized commercially available or externally prepared exosome complexes and did not isolate and extract the exosomes within their own laboratories. The study from Wan and Pamela et al. used exosomes from BLESKIN EXXO (Daeyang Medical) [16]. They were derived from lactobacillus bacteria [16]. Park et al. [11], Estabian et al. [12] and Wan et al. [17] derived their exosomes from human adipocyte conditioned media extract. Park et al. obtained their exosomes from EXO BALM by BENEV (BENEV Company Inc) [12]. Wan et al. [17] utilized stem cell‐derived exosomes from Exodew, Hyundae Meditech Co Ltd., and EXOP, Sihler Inc., Seoul Korea [17]. The Proietti et al. study [13] and Theodorakopoulou et al. [14] utilized exosomes from plant tissue. Across the studies that used externally prepared exosomes—none of the studies disclosed the regulatory status or ethical governance of the companies supplying their exosomes. Future publications should transparently describe the production methods, regulatory compliance, and ethical standards of external suppliers when exosome preparations are externally sourced.

From a regulatory standpoint, the statutory framework of human‐derived exosomes is rapidly evolving and currently the utilization of exosomes in dermatological conditions is largely unregulated [24]. Despite the benefits of exosomes and microneedling in various dermatological conditions such as melasma, there remains a lack of clear regulatory policies and guidelines across different countries and the status of exosome‐based products remains largely undefined [24].

The clinical use of exosomes presents unique regulatory challenges due to the fact there is vast diversity in exosome sources, manufacturing technologies, and purification methodologies [24]. To address these issues, regulatory frameworks must be defined to include standardized exosome extraction and purification protocols, stringent quality control, appropriate potency, stability testing and clear requirements for ethical sourcing [24]. International organizations such as the International Society for extracellular vesicles (ISEV) and the European Network on microvesicles and Extracellar vesicles in health and disease (ME‐HaD) have been created to establish baseline criteria to support clinical translation [24]. Continued liaison amongst regulatory agencies, academic institutions, and industry partners is essential to ensure safe and effective exosome therapeutic development [24]. The creation of robust frameworks and guidance will aid in promoting the clinically safe and ethically sound integration of exosome therapy into clinical practice.

## 5 Conclusion

This study highlights that more evidence is required before we can ascertain the safety and efficacy profile of microneedling and exosomes. The safety and short‐term efficacy of microneedling combined with exosomes seems promising. However, further investigations with longer follow‐up time periods and larger sample sizes are necessary to determine the long‐term safety and effectiveness of this treatment.

## Ethics Statement

The authors have nothing to report. Informed consent and ethical approval was not required as this systematic review analyzed and interpreted data from existing studies. No new studies involving human or animal tissue or participants were conducted.

## Conflicts of Interest

The authors declare no conflicts of interest.

## Data Availability

The data that support the findings of this study are available on request from the corresponding author. The data are not publicly available due to privacy or ethical restrictions.
